# Association Between Race and Blood Ferritin Level of Whole Blood Donors

**DOI:** 10.7759/cureus.82926

**Published:** 2025-04-24

**Authors:** Yamac Akgun

**Affiliations:** 1 Pathology and Laboratory Medicine, University of Southern California Keck School of Medicine, Los Angeles, USA; 2 Transfusion Medicine, Children's Hospital and Medical Center, Los Angeles, USA

**Keywords:** iron-deficiency, racial disparity, serum ferritin, transfusion medicine, voluntary blood donation

## Abstract

Background: Ferritin serves as a key marker of iron stores, and its levels are influenced by genetic, dietary, and physiological factors. However, racial disparities in ferritin levels among whole blood donors remain underexplored, despite their potential impact on donor health and transfusion practices. This study investigates racial differences in ferritin levels among whole blood donors at a single high-volume center to assess potential disparities and their implications for donor management.

Methods: A retrospective cross-sectional analysis was conducted on 26,130 whole blood donors from 2018 to 2021. Donor demographic data, including self-reported race and sex, were extracted from a centralized database. Serum ferritin levels were measured using a chemiluminescent immunoassay, and statistical comparisons were performed using Welch’s t-test and ANOVA, with a significance threshold of p < 0.05.

Results: Significant racial disparities in ferritin levels were observed. Caucasian donors exhibited the lowest ferritin levels, while Asian donors had the highest levels. African American donors displayed relatively lower ferritin levels than expected based on prior literature. Across all racial groups, males had significantly higher ferritin levels than females (p < 0.05).

Conclusion: Racial disparities in ferritin levels suggest the need for race-conscious donor health strategies, including potential pre-donation ferritin screening and tailored donation intervals to mitigate iron depletion risks. Further research is warranted to assess the impact of donation frequency on these disparities.

## Introduction

Ferritin, the primary intracellular iron storage protein, plays a critical role in maintaining systemic iron homeostasis. Clinically, serum ferritin serves as a surrogate marker of body iron stores, providing insight into iron deficiency and iron overload states [1]. In the context of blood donation, iron metabolism assumes particular importance, as frequent blood donation is associated with progressive depletion of iron stores, leading to reduced ferritin levels, latent iron deficiency, and in severe cases, anemia [2]. Consequently, understanding factors that influence ferritin levels in blood donors is paramount for ensuring donor health and the sustainability of the blood supply.

Iron metabolism is a complex physiological process modulated by genetic, nutritional, and environmental factors, as well as by sex and race [3]. It is well-documented that men generally have higher ferritin levels than women due to the absence of menstrual blood loss and hormonal differences. Similarly, race and ethnicity are increasingly recognized as variables influencing iron status, with studies showing significant variations in ferritin levels across racial and ethnic groups [4]. However, the relationship between ferritin levels and race has been underexplored in the population of whole blood donors, particularly as donor eligibility and safety criteria often fail to account for race-based disparities.

It is widely observed that individuals of African ancestry exhibit higher ferritin levels, but lower serum iron and transferrin saturation compared to individuals of European or Hispanic descent [5,6]. Multiple studies have consistently demonstrated this racial disparity, suggesting that African American populations may be at higher risk for iron deficiency, even in the absence of overt anemia [6-8]. Genetic factors likely play a key role in these differences. Polymorphisms in genes regulating iron metabolism, such as TMPRSS6, which controls hepcidin, a regulator of iron absorption, are more prevalent among individuals of African ancestry and are associated with lower iron stores [9]. In addition, variations in inflammatory markers and iron transport pathways, which differ across racial groups, may also influence ferritin concentrations [10]. For example, higher levels of inflammation in certain populations may suppress iron absorption, leading to reduced ferritin levels.

Dietary and environmental factors further compound racial differences in iron levels [11]. Lower dietary iron intake and reduced absorption efficiency contribute to lower baseline ferritin levels. Additionally, socioeconomic factors, such as disparities in access to iron-rich or fortified foods, may exacerbate the risk of iron deficiency among certain racial groups.

Populations of Asian descent tend to exhibit higher iron levels [12]. These differences may be attributed to unique dietary habits, cultural practices, and genetic factors. For example, Asian populations often demonstrate genetic variations affecting hepcidin expression and iron transporter activity, which influence iron absorption and storage [12]. Additionally, differences in dietary iron intake, such as higher consumption of plant-based iron (non-heme iron) versus heme iron, may explain the observed variability in ferritin levels [13]. Hispanic populations, on the other hand, display heterogeneity in iron status, reflecting the diverse ancestry and lifestyles within this group [14].

This study investigates the association between race and ferritin levels in whole blood donors, with the goal of identifying racial disparities in iron stores and their implications for donor safety. By exploring ferritin variability across racial groups, we aim to provide evidence-based recommendations to ensure equitable and sustainable blood donation practices. Addressing these disparities will not only protect donor health but also enhance diversity within the donor population, ultimately improving transfusion outcomes for all patients.

## Materials and methods

Materials and methods

Study Design and Population

This retrospective cross-sectional study was conducted using data from 26,130 whole blood donors who donated between 2018 and 2021. All donors included in the study donated whole blood at least once during the study period. Donors who had incomplete records or pre-existing health conditions that could affect iron metabolism (hemochromatosis) were excluded from the analysis.

Sample Collection and Preparation

Blood samples were collected prior to donation using standard venipuncture procedures. A whole blood collection kit with a diversion pouch was used to collect the initial blood sample. These samples were processed and tested within 48 hours to ensure integrity. All samples were handled following strict quality control protocols, with regular participation in external proficiency testing programs to ensure assay accuracy and reliability.

Data Collection

Data were collected from a centralized blood donation database, including donor demographic information (birth sex and self-identified race), and laboratory results. This stratification allowed for analysis of ferritin levels across sex and race and helped identify race-related trends in iron depletion.

Ferritin levels in blood donors were measured using the Beckman Coulter AU Analyzer platform (Beckman Coulter, Inc., Brea, CA), a high-throughput clinical chemistry system. This assay was performed at one of Creative Testing Solutions' (CTS) accredited laboratories. The ferritin assay employed is a chemiluminescent immunoassay (CLIA), which quantitatively measures serum ferritin concentrations as an indicator of iron stores. The measurable ferritin range in this assay was between 1 and 451 ng/mL, which provides a broad reportable range to capture both severely depleted and high ferritin levels.

Statistical Analysis

Data were analyzed using IBM SPSS Statistics software, Version 27 (IBM Corp., Armonk, NY) to assess differences in ferritin levels between the race groups. The primary variable of interest was ferritin concentration, while demographic factors were considered secondary variables. The normality of the data was evaluated using the Shapiro-Wilk test, and ferritin levels were found to follow a non-normal distribution. Therefore, non-parametric tests were used where applicable. Independent sample t-tests were employed to compare ferritin levels between each race group. Welch’s t-test was conducted for multiple pairwise comparisons. A p-value of less than 0.05 was considered statistically significant for all comparisons.

## Results

The overall analysis of ferritin levels in the donor population revealed a mean ferritin level of 85.9 ng/mL with a standard deviation of 87.7 ng/mL. The ferritin values ranged from 7 ng/mL to 451 ng/mL, with the 25th, 50th, and 75th percentiles at 29 ng/mL, 55 ng/mL, and 108 ng/mL, respectively. The donors had an average donation frequency of 1.36 times per year.

**Figure 1:**
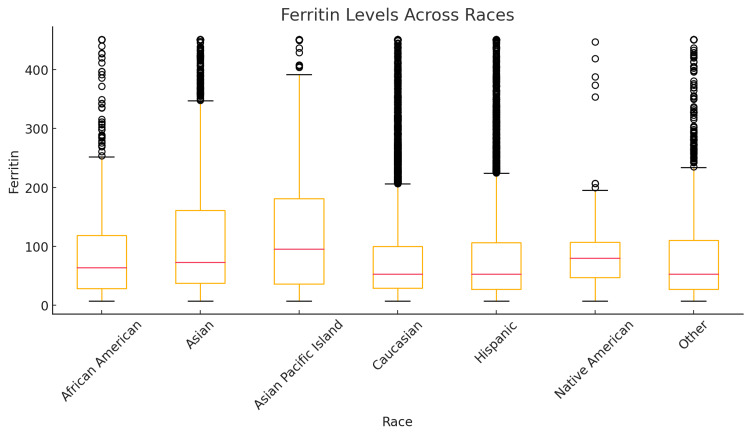
Boxplot showing the distribution of ferritin levels across different races

**Figure 2:**
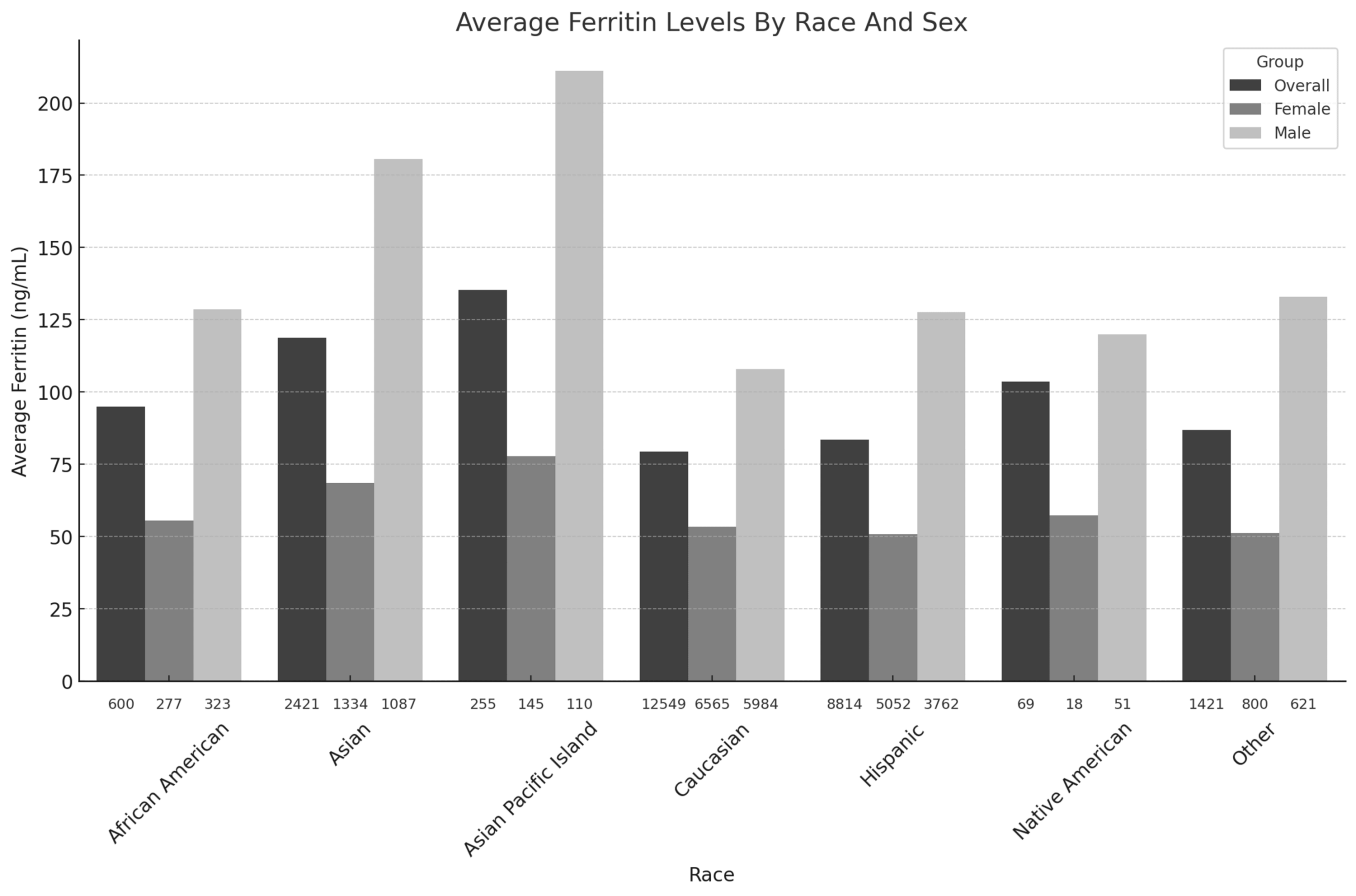
Bar graph showing the average ferritin levels by race and sex, with the sample sizes displayed below each column

Ferritin levels were analyzed across different racial groups and sexes. Figure 1 shows a boxplot showing the distribution of ferritin levels across different races. Figure 2 shows a bar graph showing the average ferritin levels for each race, broken down by overall, female, and male groups.

Sex Distribution

Among the donors, females constituted 14,189 (54.3%) of the population, while males represented 11,941 (45.7%). A comparison of ferritin levels between sexes using Welch's t-test revealed a highly significant difference (P < 0.0001), with males showing consistently higher ferritin levels compared to females.

Racial Distribution

The racial composition of the donor population showed that Caucasians comprised the largest proportion at 12,542 (48.0%), followed by Hispanics at 8,806 (33.7%) and Asians at 2,431 (9.3%). Smaller proportions were observed for other racial groups, including African Americans 601 (2.3%), Asian Pacific Islanders 261 (1.0%), and Native Americans 78 (0.3%). Donors categorized as Other constituted 1,411 (5.4%) of the population.

Ferritin Levels by Race and Sex

The analysis of ferritin levels by race and sex revealed notable differences across the donor population. Among Caucasians, the average ferritin level was 79 ng/mL overall, with males showing consistently higher values compared to females. Similarly, in the Hispanic group, ferritin levels averaged 84 ng/mL, with males exhibiting elevated levels relative to females. Asians demonstrated the highest ferritin levels among all racial groups, with an overall average of 119 ng/mL, driven primarily by the elevated levels observed in males. Asian Pacific Islanders followed closely with an average ferritin level of 135 ng/mL.

African Americans had a lower overall ferritin average of 95 ng/mL, though the male subgroup exhibited higher levels than females. Among Native Americans, ferritin levels were relatively lower, averaging 88 ng/mL, with a similar sex-based disparity favoring males. The group categorized as Other had an overall ferritin average of 107 ng/mL, highlighting variability within this group.

Comparison of Ferritin Levels Between Racial Groups

**Figure 3:**
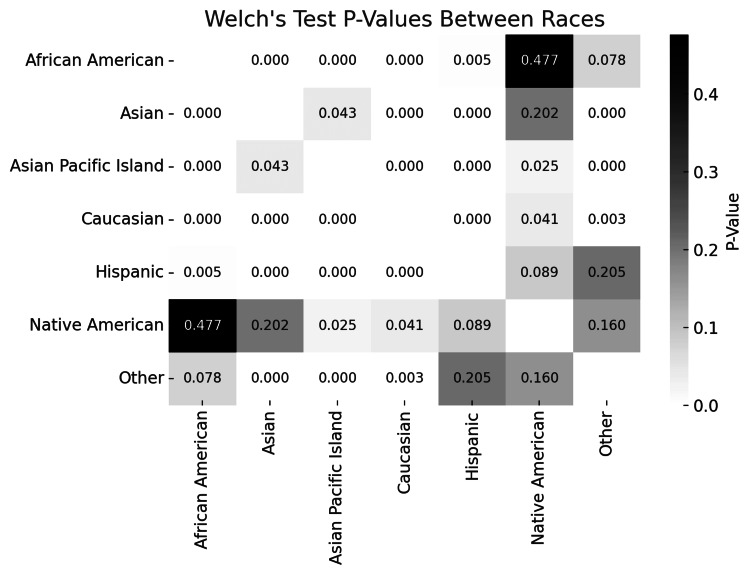
Heatmap showing Welch's test p-values between all races

Pairwise comparisons of ferritin levels between racial groups were performed using Welch's t-test. Significant differences were observed in several comparisons (Figure 3). Notably, Caucasians exhibited significantly lower ferritin levels compared to Asians and Asian Pacific Islanders. Similarly, ferritin levels in African Americans were significantly lower compared to Asians and Asian Pacific Islanders. Comparisons between Hispanics and other groups also demonstrated significant differences, particularly when compared to Asians. However, some comparisons did not reach statistical significance. For instance, the difference in ferritin levels between African Americans and Native Americans was not significant (P > 0.05).

## Discussion

This study provides novel insights into racial disparities in ferritin levels among whole blood donors, highlighting findings that, in some respects, differ from established literature [4]. Notably, Caucasian donors in our cohort exhibited the lowest ferritin levels compared to all other racial groups. This result challenges the commonly held assumption that individuals of non-Caucasian ethnic groups are more susceptible to lower iron stores, prompting a closer examination of factors that may uniquely influence ferritin levels in Caucasian donors.

Previous studies have consistently reported higher ferritin levels in African American populations [15,16]. Our findings showed that African Americans do demonstrate relatively low ferritin levels along Caucasians who displayed the lowest average ferritin levels in this cohort. This unexpected observation of both groups may reflect a confluence of demographic, environmental, and behavioral factors specific to this donor population.

One possible explanation for the lower ferritin levels observed in Caucasians could be the higher frequency of blood donation in this group. Multiple studies have demonstrated that frequent blood donors are at greater risk for iron depletion due to the cumulative loss of iron with each donation [17,18]. Historically, Caucasian populations have been overrepresented in blood donor pools, and this trend may contribute to the lower ferritin levels observed in our cohort [19]. While donation frequency was not explicitly analyzed in this study, its impact cannot be overlooked, as repeat donors are known to experience greater reductions in iron stores compared to first-time donors.

Additionally, dietary and socioeconomic factors may play a role in the observed findings. While Caucasian populations in Western countries often have access to iron-rich and fortified foods, modern dietary trends-such as reduced red meat consumption-may contribute to lower heme iron intake. The growing popularity of vegetarian and vegan diets, which are more common in certain socioeconomic and cultural subgroups of Caucasian populations, could lead to reduced iron absorption and lower ferritin levels [20]. Furthermore, differences in iron supplementation practices among racial groups may influence iron status, as individuals with lower perceived risk of deficiency may be less likely to use iron supplements.

The observed disparities also underscore the potential role of inflammation and chronic conditions in influencing ferritin levels. While ferritin primarily reflects iron stores, it is also an acute-phase reactant that can be elevated in response to inflammation or chronic disease [21]. African American populations, for instance, have been shown to exhibit higher inflammatory markers, which may artificially elevate ferritin levels despite lower actual iron stores [22]. By contrast, Caucasian donors with fewer inflammatory influences may present lower ferritin concentrations that more accurately reflect their iron status. This possibility highlights the importance of interpreting ferritin levels in the context of broader health indicators.

The higher ferritin levels observed in Asian and Asian Pacific Islander populations are consistent with prior literature and are likely influenced by genetic predispositions favoring more efficient iron absorption and storage, as well as dietary habits that include greater consumption of heme iron sources [12,13]. Similarly, Hispanic donors demonstrated intermediate ferritin levels, reflecting the heterogeneity within this population, including varying ancestries, dietary patterns, and socioeconomic conditions [14].

The findings of this study have important implications for blood donation practices. Current donor eligibility criteria, such as hemoglobin thresholds and donation frequency guidelines, do not account for racial disparities in ferritin levels. This “one-size-fits-all” approach may inadvertently place certain racial groups, such as African Americans and Caucasians, at greater risk for iron deficiency. Current donor eligibility criteria, which rely heavily on hemoglobin thresholds, may fail to identify these at-risk individuals, as hemoglobin levels do not decline until later stages of iron deficiency. Introducing pre-donation ferritin screening and implementing tailored donation intervals could help mitigate this risk, not only for Caucasian and African American donors but also for other populations at risk of iron depletion.

Moreover, these findings highlight the need to further investigate donation frequency as a confounding factor in racial disparities of ferritin levels. A more detailed analysis incorporating donation history would provide a clearer understanding of the relationship between race, donation behavior, and iron stores. Prospective studies examining longitudinal changes in ferritin levels across diverse racial groups could also elucidate the underlying mechanisms driving these disparities.

Despite the strengths of this large-scale analysis, certain limitations must be acknowledged. The retrospective design of the study limits the ability to establish causality between race and ferritin levels. Additionally, the racial categorizations used in this study may oversimplify the complex genetic and environmental factors that influence iron stores. For example, the Hispanic population is genetically and culturally diverse, and further subgroup analyses would be valuable. Moreover, while this study focused on race and ferritin levels, other factors such as dietary habits, socioeconomic status, and donation frequency were not accounted for and may have influenced the observed results.

## Conclusions

This study highlights significant racial disparities in ferritin levels among whole blood donors, with Caucasian donors exhibiting the lowest mean ferritin concentrations, contrary to prior assumptions. These findings suggest that race-specific factors, including donation frequency, dietary habits, inflammation, and genetic predispositions, may influence iron status in complex ways. Incorporating ferritin screening and tailored donation intervals into blood donor policies could enhance donor safety, particularly for populations at higher risk of iron depletion. Future studies should integrate longitudinal data and account for socioeconomic and behavioral factors to develop more equitable and evidence-based blood donation guidelines.
